# Supplementary material for: Chromothripsis during telomere crisis is independent of NHEJ, and consistent with a replicative origin
Source: Genome Res. 2019 May;29(5):737–49. doi: 10.1101/gr.240705.118 (PMC6499312; doi:10.1101/gr.240705.118)
Supplement: Supplemental Material [file supp_gr.240705.118_Supplemental_file_1.zip › contigs/annotated_contigs/DB50/contig.2.DB50_length_923_mean_cov_8.12567713976.docx]

**DB50_length_923_mean_cov_8.12567713976**

```
TATTAACACACAGTCTATTTATGCAGAAAGGCTCTTCTTTGATGTCAATGTAGGAACTTGGTAGCCTGGTCACCAGAGAGGGGTGTTCT
 >chr8:64600473-64600950 - E=5e-275
GCTTCTTTAGAAAGAATCCGTGGTTTGTCAGAAATTTCACTTGGGAAAATCAGATTTTCTTGCTTGTGTATTGCTCGATAGAAGCGATC

TTTTCTCAAACAATATGCTTTTTAATTATTTGAGGCTTAGGGACATAAACAGGCAGTTTGATGTTTGTTTGTTTGTTTGTTTGTTTGTT

TTAAAGAAATCTTAGCCCTATCTCCAAAGTAATGAGTAAAATTAGTCAGTCAGTGAATTGAGGAAGCTGCCACTTGGGACTCCAGGGGT

TGACATTTGACAGGTTTCTTAAGAGCTAGAAGAAGAAAGAAAGAAACAGGTAAACAAATGAGACTGAATGGGAGTGGAGGTGACAGGAA

AGAAGGCATGCACAGCAAAGTAATCCTCAAAA|TCCTGACCCTAAGTTCACTGGCACCGCTTTAATCACTCAGTCTTCTTGGTCTATCC
                                  >chr8:64595521-64595967 - E=2e-256
CCTGCAGACAAGCTCGTTTCAGCTTCCTCCTGACCCTGGATCACCAGCTTTCTTTGCCAACTTTGTTTTCTTCCCTTTCAATTCATCTA

GTCCAGTGACCCAGCAGTCTCCTACAGCTGCCCAAGTACCTTTCTTAGGGTAAAGGCTCAGGGACTATTTGTTGAATAAATTAATAATT

AACTATCTTATAATGATTTTAACACCCATTAGCTTAAAGCAATTATCATCTTTTGGATATTTTCTATATTTTAATGAATGTAATTTTAA

ACTCTTCTTGATAAGGGAAAATTTATTAGTTTAAGAGCTTTCAAACATTACATTTGTACTGTTTAATTCTGAGTCAAAGAACGTGCTTA

GAAGTCAGGCTTAAAAGGCTGTCTGGGGTAAACA
```
